# Does the Stage of University Education Differentiate Midwifery Students in Terms of Their Behaviors in Certain Situations and Sense of Self-Efficacy?

**DOI:** 10.3390/ijerph191811427

**Published:** 2022-09-10

**Authors:** Grażyna J. Iwanowicz-Palus, Justyna J. Krysa, Agnieszka Palus, Mateusz Cybulski, Magdalena Korżyńska-Piętas, Agnieszka Bień

**Affiliations:** 1 Faculty of Health Sciences, Medical University of Lublin, 4–6 Staszica St., 20-081 Lublin, Poland; 2 Medical Center in Nowy Dwór Mazowiecki, 2 Miodowa St., 05-100 Nowy Dwór Mazowiecki, Poland; 3 Department of Integrated Medical Care, Faculty of Health Sciences, Medical University of Bialystok, 7A Marii Skłodowskiej-Curie St., 15-096 Bialystok, Poland

**Keywords:** midwifery, midwifery students, education, student behaviors, self-efficacy

## Abstract

Midwifery students’ behaviors in relevant spheres of their lives, as well as their sense of self-efficacy, can affect the process of training in the midwifery profession. The aim of the study was to determine the behaviors of students in Poland, assessed in a situational context, as well as their sense of self-efficacy in correlation with these behaviors at different levels of education in the midwifery profession. The study group included first- and third-year bachelor’s degree (BS) midwifery students, as well as master’s degree (MS) midwifery students. The survey was conducted on 1031 students. The ‘Inventory for Personality Assessment in Situations’ (IPS) and the General Self-Efficacy Scale (GSES) were used in the study. The largest group of students that were categorized as having problematic profiles was observed in the social-communicative domain, which indicates the necessity of introducing corrective and therapeutic actions concerning their interpersonal relations. The leading trait in the social-communicative domain among the BS students was sensitivity to frustration. The lowest self-confidence was observed among the third-year BS students. The average result of generalized self-efficacy among all the respondents was M = 28.36 (SD = 4.41), which indicates the average level of the obtained results. Students at different stages of midwifery programs demonstrate different behaviors when assessed in the situational context.

## 1. Introduction

The education of nurses and midwives in Poland is provided in accordance with the guidelines established by the WHO European Strategy for Nursing and Midwifery Education from 1999. The standards of educating Polish midwives have been adjusted to the EU requirements for regulated professions, defined in the EU directives on the recognition of professional qualifications of midwives [1,2,3]. Future midwives in Poland are educated at the university level, in accordance with the Law on Higher Education and Science, the Act on the Professions of Nurses and Midwives, and other legal acts that set educational standards [4,5,6,7].

In accordance with the Bologna declaration of 1999, midwifery education is divided into two cycles. The first cycle lasts six semesters. The minimum number of educational hours is 4720, including at least 2300 hours of practical education. Graduates of the first cycle receive a bachelor’s degree (BS) in midwifery and can apply for admission to the second cycle. Graduates of the second cycle receive a master’s degree in midwifery (MS). MS studies last four semesters and the minimum number of theory and practical classes is 1300 [2,4,8]. Midwifery education is challenging, dynamic, and intensive. Thus, midwifery students might feel anxious, lost, and doubtful about their ability to cope with their role as a student and, later, as a midwife. Additionally, the socio-cultural environment generates certain, rather demanding, requirements towards the students. Long hours of studying, a lack of free time, exams, and heavy workload are common characteristics for the learning process in university education, and all these require the student to significantly activate not only the mental but also the physical sphere. If appropriate strategies of coping with difficult conditions are not implemented in time, the situation may exacerbate, possibly leading to occupational burnout at the very beginning of one’s professional development. Thus, midwifery education and the profession of a midwife require discussions regarding the complexity of the behaviors displayed by young people in certain situations. The requirements imposed on midwifery students concern mainly their education, work life, and leisure time activities [8,9].

The aim of the study was to determine the behaviors of students in Poland, assessed in a situational context, as well as their sense of self-efficacy in correlation with these behaviors at different levels of education in the midwifery profession.

## 2. Materials and Methods

### 2.1. Study Groups

The study was conducted from October 2016 to September 2018 among bachelor’s degree (BS) and master’s degree (MS) midwifery students at Polish universities (8 out of 15 randomly chosen universities offering first-cycle and second-cycle degree programs in midwifery).

In the 2015/2016 academic year, 4012 students studied midwifery in Poland, including 2800 students at the bachelor level (first year of study—1007, second year of study—996, third year of study—797) and 1212 students at the master level (first year of study—633, second year of study—579). The survey was conducted from October 2016 to September 2017 among students of randomly selected universities in Poland educating in the field of midwifery at the bachelor and master levels. The 8 centers from which the students participating in the study came were drawn, i.e.,: the Medical University of Gdansk, Pomeranian Medical University in Szczecin, Silesian Medical University in Katowice, Jan Kochanowski University in Kielce, the Medical University of Bialystok, the Medical University of Lublin, Wroclaw Medical University, and the University of Rzeszów.

The participants were first-year BS students (in the 2nd semester, the students start their practical classes and internships, which allows them to verify their ideas of this profession), third-year BS students (in the 6th semester, the students have completed most of the BS program and are about to take the final exam and choose which professional path to follow next), and MS students (who already work in the profession, are starting families, trying to reconcile their professional and family lives, or are about to make a decision regarding which professional path to follow next). The first- and second-year MS students were classified in one group, as the differences between them are not as pronounced as those between BS students. In total, 1300 questionnaires were distributed among the respondents, of which 1031 questionnaires were received that had been completed correctly and which qualified for further analysis. The return rate was 79.31. The questionnaires were sent and received by post. The questionnaires were accompanied by an addressed return envelope and stamp. The surveys were distributed to students by university teachers who agreed to cooperate. Among the correctly completed questionnaires, 350 belonged to first-year bachelor midwifery students, 358 correctly completed questionnaires belonged to third-year bachelor students, while 323 belonged to second-year master students. Among the correctly completed questionnaires, including 350 first-year BS students, 358 third-year BS students, and 323 MS students.

Based on Resolution no. KE-0254/143/2016, the study was approved by the Bioethical Committee at the Medical University of Lublin. The respondents were informed that the study was voluntary and anonymous, and that the results would be used solely for scientific purposes. The study was conducted in accordance with the latest version of the Declaration of Helsinki.

### 2.2. Assessments

The study used a diagnostic survey with questionnaires. The tool applied was the IPS—Inventory for Personality Assessment in Situations (Schaarschmidt, Fischer, 1999; Rongińska, 2005), the GSES—Generalized Self-Efficacy Scale–(Schwarzer, Jerusalem, Juczyński), and a standardized interview questionnaire comprising questions on the participants’ characteristics [10,11,12,13].

In the IPS questionnaire, respondents self-assess their behaviors and experiences concerning the demands of their everyday life. The questions pertain to behaviors in three domains:

- The social-communicative domain, assessing interpersonal competences, teamwork skills, and the ability to solve conflicts;
- The achievement domain, assessing respondents’ approach towards achieving goals and their ability to adjust to work-related changes (vocational education), take risks, and face complex tasks;
- The health and recreation domain, assessing respondents’ behaviors in their free time, the ability to relax (also actively), and their approach to preventive healthcare [10,11].

The main goal of the IPS questionnaire is to define behavioral patterns in these domains, whereas the main goal of the present study was to identify mutual relations between its individual scales. This allowed for creating individual, specific profiles of the participants or groups studied which reflect characteristic behavior patterns in the pre-defined situations. Following a cluster analysis, the authors of the tool distinguished six profiles in the social-communicative area (domain A) and the achievement area (domain B), and five behavior profiles in the health and recreation area (domain C) [10,11].

The IPS questionnaire allows for identifying problematic areas of behavior and presenting a program of preventive and therapeutic activities. It consists of 15 situations with 5–9 statements assigned to them that describe the behaviors of an individual in a given situation. Using a four-point scale, respondents mark to what extent a given behavior is true for them: 1—not true at all, 2—not really true, 3—fairly true, 4—definitely true. The assumption of the tool is that the raw data are first analyzed for a given group and then normalized mean values are calculated for individual IPS scales. The raw data are standardized using a 9-point scale with the extreme scores of 1 and 9 and an average of 5. Scores that exceed the average (0.5) are of particular importance, as they indicate that some intervention might be needed. Traits that go furthest from the average are considered dominating behavior domains [10,11]. The IPS results can be interpreted based on a score analysis for each scale. The IPS by Schaarschmidt and Fischer has been adapted for use in Polish settings by Rogińska. The reliability of the IPS questionnaire was assessed, with Cronbach’s alpha ranges between 0.61 and 0.92. The present study showed the questionnaire had the following reliability: social-communicative domain: 0.680-0.883, achievement domain: 0.729–0.827, and health and recreation domain: 0.703–0.807 [10,11].

The Generalized Self-Efficacy Scale (GSES) by Schwarzer and Jerusalem, adapted for use in Polish settings by Juczyński, is directed to adults, both healthy and sick. The scale consists of 10 questions. Each question has four answer options, of which only one can be selected. The response scale is as follows: 1—no, 2—rather no, 3—rather yes, 4—yes. This allows us to measure the strength of an individual’s overall belief in the effectiveness of coping with obstacles and difficult situations on a daily basis. The sum of all scores gives an overall self-efficacy index. The minimum value is 10, and the maximum is 40 points. The higher the assigned score, the higher the sense of self-efficacy. The results should be interpreted in relation to sten norms. A score of 1 to 4 sten is defined as low, a score of 7 to 10 sten is high, while scores within 5 and 6 sten are considered average. The reliability of the questionnaire, as measured by Cronbach’s internal consistency coefficient α, is 0.85 [12,13].

### 2.3. Statistical Analyses

The data were analyzed using the statistical package SPSS Statistics (v. 21) (Predictive Solutions Sp. z o. o., Kraków, Poland). The quantitative variables were described with a mean (M) and standard deviation (SD). Normality was verified with the Shapiro–Wilk test. The qualitative variables were provided as numeric and percentage values. The chi-square test (χ^2^) was used to determine correlations between qualitative variables. When the requirements set for parametric tests were satisfied (quantitative variables), a univariate analysis of variance ANOVA (F) was used for independent groups, whose aim was to verify whether the means of the variables analyzed were equal in several populations. The significance level used in the study was *p* < 0.05.

## 3. Results

The study was conducted among 1031 midwifery students. Of these, 350 were first-year bachelor students, 358 were third-year bachelor students, and 323 were second-year master students.

### 3.1. Characteristics of the Midwifery Students

In the studied group of BS students, the majority were people who were under 24 years of age (1st year—96.2%, 3rd year—89.1%), unmarried (1st year—99.1%, 3rd year—96.6%), living in the city (1st year—58.3%, 3rd year—71.8%) and not professionally active (1st year—98.9%, 3rd year—93.3%). On the other hand, the group of MS students studied was predominantly older than 24 years old (62.2%) and professionally active (56.3%), but similarly (*p* > 0.05) unmarried (81.4%) and living in the city (68.1%), as was the case for BS students (Table 1).

### 3.2. An Overview of IPS Scale Values concerning Polish Midwifery Students on Three Relevant Life Domains in Situational Contexts

Table 2 presents the IPS scale scores for Polish midwifery students in three life domains in their situational context. The dominating trait of the BS students in the social-communicative domain was sensitivity to frustration—A6 (group I–M = 5.41; group II–M = 5.36), whereas among the MS students, this was a tendency to engage in confrontation in social conflict situations—A3 (M = 5.37). The analysis of the social-communicative scales revealed statistically significant differences (*p* < 0.001) in sensitivity to frustration (scale A6) among the groups studied. Even though all the groups scored above the average of 5.0 (group I–M = 5.41; group II–M = 5.36; group III–M = 5.10), indicating that the midwifery students are characterized by emotional overload, despondency, and self-absorption (Table 1 and Table 3), these traits were most dominant among the first-year BS students. The higher the year of study, the lower the scores concerning sensitivity to frustration.

The analysis of scores in the achievement scales demonstrated statistically significant (*p* = 0.021) differences between the midwifery students in terms of commitment when a high level of performance is required (scale B1). The higher the level of education of the respondents, the lower their commitment when a high level of performance is required. The BS students scored above the mean (group I–M = 5.20; group II–M = 5.03), which demonstrates that the majority of the students in this group are ready to make an effort and act quickly. On the other hand, the MS students (M = 4.85) avoided effort and were reserved (Table 1 and Table 3).

The dominating trait of the BS students in the achievement domain was low self-confidence in test situations—B4 (group I–M = 4.53; group II–M = 4.40). At the same time, they were ready to take risks and develop their professional careers—B5 (group I–M = 5.20; group II–M = 5.11). On the other hand, the dominating trait of the MS students in this domain was a low level of optimism in the face of everyday demands—B6 (M = 4.70).

Even though all the groups scored below the average (5.0) in the B4 scale, which indicates that the students are unsure, timid, irritable, and unstable (group III and group I), statistically significant differences between the groups (*p* = 0.048) show that these traits are less characteristic of MS students (M = 4.74) in comparison to third-year BS students (M = 4.40).

At the same time, the analysis showed that the dominating trait in all groups of midwifery students regarding the health and recreation domain was the low value of health prevention when warning signals appear—C3 (group I–M = 4.59; group II–M = 4.48; group III–M = 4.53), which proves that the students are negligent, careless, and undisciplined when health prevention in a personal context is concerned.

Over one-third of the first-year BS students (37%), more than a half of the third-year BS students (59%), and more than two-fifths of the MS students (43%) represented the problematic profiles (AP3, AP4, AP5) in the social-communicative domain. The AP3 profile, requiring corrective measures in order to lower the expansion of the undesirable traits and increase the quality of interpersonal relations, was twice as common in the group of third-year BS students (22%) than among first-year BS students (8%) or MS students (9%). These proportions were reversed for the “unfavorable” AP6 profile, requiring corrective measures with regard to a wide scope of interpersonal skills (group I = 18%; group II = 5%; group III = 19%). These differences were statistically significant (*p* < 0.001), as shown in Table 3.

### 3.3. The Midwifery Students’ Assessment of Self-Efficacy

The average scores of self-efficacy between different years of students were very similar. In the group of first-year bachelor students, the mean was M = 28.34 (SD ± 4.65); in the group of third-year bachelor students, the means was M = 28.33 (SD ± 4.44); while in the group of master students, the mean was M = 28.42 (SD ± 4.11). Comparing the generalized sense of self-efficacy in all study groups, there was no statistically significant difference in the level of self-efficacy between the different groups of midwifery students (*p* > 0.05). The mean score of generalized sense of self-efficacy among all the students studied was M = 28.36 (SD = 4.41), as shown in Table 4.

The correlation analysis between generalized sense of self-efficacy and the dominant behaviors of I and III bachelor students assessed in a situational context showed a statistically significant (*p* < 0.001) positive correlation of weak strength between the studied characteristics. The higher the generalized sense of self-efficacy of I bachelor students, the higher their sensitivity to frustration (Group I: r = 0.264; Group II: r = 0.253), the higher their self-confidence in situations of exam demands (Group I: r = 0.123; Group II: r = 0.256) and the better their adherence to health prevention in relation to warning signals (Group I: r = 0.181; Group II: r = 0.269), as shown in Table 5.

The correlation analysis between the generalized sense of self-efficacy and the dominant behaviors of master students assessed in a situational context showed a statistically significant (*p* < 0.001) positive correlation of weak strength between the studied characteristics. The higher the sense of self-efficacy, the more often II master students tended to be confrontational in situations of social conflict (r = 0.263), the more often they showed optimism in the face of daily demands (r = 0.165), and the more often they carried out health prevention in case of warning signals (r = 0.279), as shown in Table 6.

## 4. Discussion

The results of the present study allowed for identifying and differentiating various behaviors of midwifery students in Poland, as assessed in various situations, and their sense of self-efficacy. Effective communication in healthcare determines the right course of treatment, increases the quality of care, and enhances patients’ satisfaction [14]. The ability to communicate in an assertive way is the key to establishing safe and effective teamwork [15,16]. Midwifery programs require students to develop communicative abilities [4,5,17,18]. In 2018, Suikkala et al. [19] indicated that the real contact of students with patients is vital for developing the skills that will later be necessary in their job-related activities.

Based on the study results, most of the first-year BS students were assigned to AP2 (an average profile), which described people who can successfully manage social and communicative situations while experiencing successes and failures in this domain. They do not require special support since they are able to deal satisfactorily with various issues in this area. It is worth mentioning that the midwifery program for the first-year BS students in Poland includes psychology and interpersonal communication as an obligatory subject, which allows them to become familiar with the models and styles of appropriate communication [6,7]. However, over one-third of the first-year BS students, more than a half of the third-year BS students, and more than two-fifths of the MS students were assigned to the problematic profiles (AP3, AP4, AP5) in the social-communicative domain. Such students require corrective actions and therapy aimed towards increasing the quality of their interpersonal relations, which will help them deal with difficulties in such relations and strengthen the sense of their own “selves”.

Alimoradi et al. [20] and Sanders et al. [21] advocated for the introduction of a separate course on communicative skills into the midwifery studies curriculum. Moreover, Santos et al. [22] also indicated that there is a need to develop interpersonal competencies among BS nursing students in Brazil. In Denmark, there are workshops organized for medical staff that improve the quality of information exchange among colleagues and enhance communication with the patient [23].

In the present study, the leading trait in the social-communicative domain among the BS students was sensitivity to frustration. The inclination towards frustration might lead to professional burnout. It is a response to chronic and intense stress connected to students’ roles and the situational context in which they function. It has been proven that different leisure activities can protect against professional burnout [9]. The achievement domain demonstrates whether midwifery students are able to react proactively in task-oriented situations. The students need to be aware that this profession requires life-long learning and constant development, as reflected in the achievement domain [4].

In all the groups studied, regardless of the stage of education, most of the students were categorized with “problematic” or “unfavorable” profiles in the achievement domain. Such students require corrective actions which will help them to learn active strategies for solving problems at work and to develop task-oriented competencies. They also require therapy-based activities in order to develop the ability to deal with stress. The research conducted on Malaysian students of various majors by Elias et al. [8] indicates that medical students, mainly those in the final year of their studies, were most subjected to stress. What might be worrying is that the higher the level of stress they experienced, the lower the academic results they achieved [8]. In a study from 2017 by Saini et al. [24], nursing students most often named fear of bad grades, their parents’ expectations, fewer breaks, and study overload as their greatest stressors. Skodova et al. [25] believe that nursing and midwifery students can be helped to cope with stressful situations by way of training programs on how to manage stress, gain communicative and interpersonal skills, cooperate, and make group decisions.

In the present study, BS students proved to be ready to put in effort and act quickly. On a daily basis, they are committed to and ready for change. In this respect, they demonstrate a proactive approach to reality. Power [26] demonstrated that third-year midwifery students have significantly higher expectations towards their professional practice. Carolan and Kruger [27], in 2011, postulated the necessity of providing greater support to BS students. Such support helps students adapt to university life. Midwifery studies should also include strategies involving personalized activities in their curricula and programs that will support the students when they move from their student role to professional practice [28].

The present study showed that the dominating trait among the BS students in the achievement domain was low self-confidence in test situations, and among MS students the dominant trait was low optimism in the face of everyday demands. Taheri et al. [29], in 2018, presented slightly different results concerning optimism among midwifery students. Most of them had a relatively good level of optimism. The literature on this subject suggests that optimism has a positive impact on mental health and mood. Optimists tend to be more successful than pessimists. This trait also helps individuals achieve success in life and increases one’s resistance to stress [30]. Nursing and midwifery teachers have to be aware of this impact and provide appropriate support to students, both in the clinical and academic environment [31,32].

In the health and recreation domain, all the groups studied presented low scores for health prevention when warning signals appear, which proves that the students are negligent, careless, and undisciplined where their own health prevention is concerned. Therefore, it seems advisable to intensify the promotional and educational activities targeted at students, motivating them to be more physically active. It is important to implement educational and supportive programs aimed at developing young people’s ability to choose and lead a healthy lifestyle and use thought-through, effective methods of coping with problems.

Polish students show an average level of self-efficacy. These results do not correspond with the results obtained by Gudayu et al. (2015) in a study of midwifery students from Gondar University in Ethiopia, as well as the results of Kulik et al. (2016) in a study of Polish female students of uniform master’s degree programs in various fields of study, where students obtained a high level of the indicated trait [33,34]. The results of Kot et al. (2017) and Żołnierz et al. (2017) indicate that students from higher years of study showed higher levels of self-efficacy [35,36]. In contrast, both Moattari et al. (2013) and Sohrabi et al. (2016) showed no effect from the educational stage of Iranian midwifery students on their sense of self-efficacy [37,38]. In medical professions, a higher sense of self-efficacy is desirable. It influences the choice and decision to undertake an activity, as was demonstrated in a study by Andruskiewicz et al. (2011) [39]. Self-efficacy differentiates people in terms of thinking, feeling, and acting. Higher self-confidence triggers additional energy in people and makes them engage more strongly in the intended tasks, even in the face of failure. As Juczyński (2012) points out, if the sense of self-efficacy plays a regulatory role, an individual must take into account his actual abilities. Excessive optimism usually leads to disappointment [15]. This may explain the results obtained in the author’s own research, including the susceptibility to frustration of the studied groups, which increases with the increase in the sense of self-efficacy.

The results of this study indicate that a higher generalized sense of self-efficacy of students in all study groups determines the achievement of higher health prevention scores for warning signs. As indicated by Zarzeczna-Baran and Wojdak-Haasa (2007) in a study conducted in medical students, a high level of knowledge about health-promoting lifestyles did not always translate into positive health behaviors [40]. A study conducted by Alghamdi (2021) showed that medical students have a satisfactory level of knowledge about a pro-healthy lifestyle but do not lead a healthy lifestyle on a daily basis, which needs to be taken care of [41]. As Brehm et al. (2016) indicate, students’ clinical measures and lifestyle behaviors remain generally healthy throughout medical school, yet some students exhibit cardiometabolic risk and diet and activity habits that do not align with national recommendations. Curricula that include personal health and lifestyle assessment may motivate students to adopt healthier practices and serve as role models for patients [42].

## 5. Conclusions

Students at different stages of midwifery programs demonstrate different behaviors when assessed in the situational context. The largest group of students who were categorized with problematic profiles was observed in the social-communicative domain, which indicates the necessity of introducing corrective and therapeutic actions concerning their interpersonal relations. The leading trait in the social-communicative domain among the BS students was sensitivity to frustration. Moreover, the higher the year of midwifery studies, the lower the student’s commitment when a high level of performance is required.

The IPS questionnaire allowed for a precise identification of deficit areas among specific groups of Polish midwifery students. It is advisable to provide support in the process of student adaptation, mainly in the first year of studies. The introduction of additional courses on communicative skills for midwifery students of various years is advocated. It is important to implement educational and support programs for midwifery students regarding the use of effective methods for coping with problems.

The analysis of the relationship between the generalized sense of self-efficacy and the dominant behaviors of the midwifery students, assessed in a situational context, showed that the higher the generalized sense of self-efficacy of the I student, the higher their sensitivity to frustration, the higher their self-confidence in situations of exam demands, and the better their health prevention in case of warning signals. However, the higher the IIº student’s sense of self-efficacy, the more confrontational they tended to be in situations of social conflict, the more often they showed optimism in the face of daily demands, and the more often they carried out health prophylaxis in response to warning signals.

## 6. Strengths and Weaknesses of the Study

The use of the IPS tool for research into midwifery students at different stages of their education is the strength of the study. The tool not only allows problems and deficit areas to be diagnosed, but also the implementation of optimal preventive measures. The inability to compare and contrast the results of this pioneering research with those of other authors, which are not yet available, is the weakness of our study.

## Figures and Tables

**Table 1:** The characteristics of the midwifery students who took part in the study.

Characteristics of the Group		1st Group: 1st-Year BS Students N (%)	2nd Group: 3rd-Year BS Students N (%)	3rd Group: MS Students N (%)
		350 (100)	358 (100)	323 (100)
Age	Up to 21 y/o	312 (89.1)	51 (14.2)	1 (0.3)
	22–23 y/o	25 (7.1)	268 (74.9)	121 (37.5)
	More than 24 y/o	13 (3.7)	39 (10.9)	201 (62.2)
Relationship status	Single	347 (99.1)	346 (96.6)	263 (81.4)
	Married	3 (0.9)	12 (3.4)	60 (18.6)
Residence	City	204 (58.3)	255 (71.8)	220 (68.1)
	Country	146 (41.7)	103 (28.2)	103 (31.9)
Professional status	Not professionally active	346 (98.9)	334 (93.3)	141 (43.7)
	Professionally active	4 (1.1)	24 (6.7)	182 (56.3)

BS—Bachelor of Science; MS—Master of Science.

**Table 2:** An overview of IPS Scale Values Concerning Polish Midwifery Students on Three Relevant Life Domains in Situational Contexts.

Behavior Domain	IPS Scale	Behavior Domain	1st Group: 1st-Year BS Students		2nd Group: 3rd-Year BS Students		3rd Group: MS Students		Statistical Analysis	
			M	SD	M	SD	M	SD	F	*p*
Social-communicative domain	A1 scale	Activity in familiar communicative situations	5.08	1.80	5.16	1.89	4.89	1.79	1.931	0.146
	A2 scale	Assertiveness when communication is required	4.96	2.05	4.93	2.03	4.80	1.90	0.603	0.547
	A3 scale	Tendency for confrontation in social conflict situations	5.17	1.60	5.28	1.57	5.37	1.55	1.365	0.256
	A4 scale	Efficacy in a managing role	4.74	1.71	4.84	1.69	4.67	1.51	2.515	0.081
	A5 scale	Considerateness in social responsibility situations	5.07	1.87	5.06	1.74	4.94	1.70	0.554	0.575
	A6 scale	Sensitivity to frustration	5.41	1.98	5.36	1.99	5.10	1.80	8.988	0.001
Achievement domain	B1 scale	Commitment when a high level of performance is required	5.20	1.67	5.03	1.57	4.85	1.64	3.890	0.021
	B2 scale	Tendency of inflexibility when requirements change	4.78	1.83	4.87	1.73	4.80	1.55	0.269	0.764
	B3 scale	Stability when under stress	4.99	1.69	4.83	1.60	4.90	1.52	0.881	0.415
	B4 scale	Self-confidence in test situations	4.53	1.86	4.40	1.82	4.74	1.73	3.043	0.048
	B5 scale	Readiness to take risks and pursue a professional career in conditions of difficult job-related challenges	5.20	1.82	5.11	1.76	5.10	1.66	0.341	0.711
	B6 scale	Optimism in the face of everyday demands	4.66	1.87	4.58	1.65	4.70	1.66	0.427	0.653
Health and recreation domain	C1 scale	Ability to relax after the working day	4.62	1.77	4.56	1.67	4.56	1.65	0.144	0.866
	C2 scale	Active recreation behavior in free time	4.90	2.15	4.88	2.01	5.00	2.17	0.310	0.733
	C3 scale	Preventive health behavior in response to warning signals	4.59	1.81	4.48	1.75	4.53	1.69	0.349	0.705

BS—Bachelor of Science; MS—Master of Science; IPS—Inventory for Personality Assessment in Situations; M—Mean; SD—Standard Deviation; F—univariate analysis of variance ANOVA.

**Table 3:** Division of the midwifery students in accordance with the behavior profiles in the domains studied.

Behavior Domain	Behavior Profiles of the Respondents	1st Group: 1st-Year BS Students n (%)	2nd Group: 3rd-Year BS Students n (%)	3rd Group: MS Students n (%)
Social-communicative domain	AP1 profile (optimal profile)	44 (12.6)	100 (27.9)	29 (9.0)
	AP2 profile (average profile)	114 (32.6)	30 (8.4)	93 (28.8)
	AP3 profile (“problematic” profile)	28 (8.0)	77 (21.5)	29 (9.0)
	AP4 profile (“problematic” profile)	62 (17.7)	57 (15.9)	71 (22.0)
	AP5 profile (“problematic” profile)	38 (10.9)	78 (21.8)	38 (11.8)
	AP6 profile (“unfavorable” profile)	64 (18.3)	16 (4.5)	61 (18.9)
	Undetermined ¹	0 (0.0)	0 (0.0)	2 (0.6)
	χ^2^ = 183.868; df = 1; *p* = 0 < 0.001			
Achievement domain	BP1 profile (optimal profile)	36 (10.3)	35 (9.8)	19 (5.9)
	BP2 profile (average profile)	109 (31.1)	99 (27.7)	99 (30.7)
	BP3 profile (“problematic” profile)	42 (12.0)	27 (7.5)	39 (12.1)
	BP4 profile (“problematic” profile)	50 (14.3)	55 (15.4)	57 (17.6)
	BP5 profile (“problematic” profile)	83 (23.7)	102 (28.5)	89 (27.6)
	BP6 profile (“unfavorable” profile)	27 (7.7)	37 (10.3)	18 (5.6)
	Undetermined ¹	3 (0.9)	3 (0.8)	2 (0.6)
	χ^2^ = 17.601; df = 12; *p* = 0.128			
Health and recreation domain	CP1 profile (optimal profile)	71 (20.3)	63 (17.6)	65 (20.1)
	CP2 profile (average profile)	103 (29.4)	104 (29.1)	96 (29.7)
	CP3 profile (“problematic” profile)	48 (13.7)	57 (15.9)	42 (13.0)
	CP4 profile (“problematic” profile)	34 (9.7)	46 (12.8)	29 (9.0)
	CP5 profile (“unfavorable” profile)	94 (26.9)	88 (24.6)	91 (28.2)
	Undetermined ¹	0 (0.0)	0 (0.0)	0 (0.0)
	χ^2^ = 5.609; df = 8; *p* = 0.691			

BS—Bachelor of Science; MS—Master of Science. ¹ The “undetermined” position refers to those respondents who could not be assigned to one specific profile at the level of 95% probability while maintaining a defined criterion.

**Table 4:** The midwifery students’ assessment of self-efficacy.

GSES	M	SD	Min	Max	Me	Sten	Statistical Analysis	
							F	*p*
1st group: 1st-year BS	28.34	4.65	14.00	40.00	29.00	6	0.046	0.955
2nd group: 3rd-year BS	28.33	4.44	11.00	40.00	29.00	6		
3rd group: MS students	28.42	4.11	12.00	40.00	29.00	6		
In total	28.36	4.41	11.00	40.00	29.00	28.36		

F—univariate analysis of variance ANOVA; BS—Bachelor of Science; MS—Master of Science.

**Table 5:** The sense of generalized self-efficacy in correlation with dominant behaviors assessed in the situational context of I and II year bachelor midwifery students.

Dominant Behaviors of Bachelor Midwifery Students	GSES			
	1st Group–1st-Year BS		2nd Group 3rd-Year BS	
	r	*p*	r	*p*
A6—sensitivity to frustration	A: the sphere of social-communicative behavior			
	0.264	<0.001	0.253	<0.001
B4—confidence in situations of exam demands	B: sphere of achievements			
	0.123	0.022	0.256	<0.001
C3—preventive health care after warning signs	C: the sphere of health and relaxation behavior			
	0.181	0.001	0.269	<0.001

R—Pearson’s r; *p*—relevance; GSES—Generalized Self-Efficacy Scale; BS—Bachelor of Science.

**Table 6:** The sense of generalized self-efficacy in correlation with dominant behaviors assessed in the situational context of midwifery students.

Dominant Behaviors of Master Midwifery Students	GSES	
	3rd Group–Master Students	
	r	*p*
A3—tendency to confront in situations of social conflict	A: the sphere of social-communicative behavior	
	0.263	<0.001
B6—optimism in the face of daily demands	B: sphere of achievements	
	0.165	<0.001
C3—preventive health care after warning signs	C: the sphere of health and relaxation behavior	
	0.279	<0.001

R—Pearson’s r; *p*—relevance; GSES—Generalized Self-Efficacy Scale.

## Data Availability

Not applicable.
